# A Randomized Controlled Trial for the Effectiveness of Aromatherapy in Decreasing Salivary Gland Damage following Radioactive Iodine Therapy for Differentiated Thyroid Cancer

**DOI:** 10.1155/2016/9509810

**Published:** 2016-11-30

**Authors:** Michihiro Nakayama, Atsutaka Okizaki, Koji Takahashi

**Affiliations:** Department of Radiology, Asahikawa Medical University, Asahikawa, Japan

## Abstract

*Objective.* The aim of this study was to investigate effects of aromatherapy in decreasing salivary gland damage for patients undergoing radioactive iodine (RAI) therapy with differentiated thyroid cancer (DTC).* Materials and Methods.* The subjects were 71 patients with DTC. They were divided into aromatherapy group (group A, *n* = 35) and a control group (group B, *n* = 36). We blended 1.0 mL of lemon and 0.5 mL of ginger essential oils. The patients in the inhalation aromatherapy group inhaled this blend oil and those in the control group inhaled distilled water as placebo for 10 min during admission. We statistically compared salivary gland function before and after treatment between groups A and B.* Results.* In comparison with group B, the rate of change of the accumulation rate was significantly higher in the parotid glands and submandibular glands of group A (*P* < 0.05). In comparison with group B, a significant increase in rate of secretion change before and after treatment was noted in the bilateral parotid glands in group A (*P* < 0.05).* Conclusion.* Because an amelioration of salivary gland function was observed in the present study, our results suggest the efficacy of aromatherapy in the prevention of treatment-related salivary gland disorder. This trial is registered with UMIN Clinical Trial Registry: UMIN000013968.

## 1. Introduction

The incidence of thyroid cancer has been increasing worldwide over the past few decades. An estimated 64,300 new cases of thyroid cancer are expected to be diagnosed in the United States in 2016, with 3 of 4 cases occurring in women [1].

The initial treatment for a majority of differentiated thyroid cancer (DTC) patients is total thyroidectomy [2]. Radioactive iodine (RAI) therapy is an effective treatment for relapse or metastasis after DTC surgery and significantly improves the prognosis of recurrent DTC [3, 4].

The most common long-term complication of RAI therapy after thyroidectomy is salivary gland dysfunction. Saliva lubricates the oral mucosa, which allows proper speaking, swallowing, and tasting [5]. The prevalence of chronic RAI sialadenitis, an inflammation of the salivary glands, ranges from 11% to 67% after RAI therapy [6–11]. This condition causes pain and swelling which lead to oral discomfort [5, 7].

Aromatherapy using essential oils to bring about various psychological and physical effects has attracted considerable attention in recent years [12–14]. It is administered in various ways, including through massage, bathing, and inhalation. On inhaling essential oils, olfactory receptor cells are stimulated and impulses are transmitted to the limbic system, the center of autonomic function and emotions. The properties of oils, their fragrances, and their effects determine the level of stimulation of this system [15]. Olfactory information is further transmitted to primary olfactory regions in the brain, most of which are components of or strongly connected to the limbic system [16, 17].

In general, the efficacy of aromatherapy is primarily based on empirical evidence reported through folk remedies, and only few effects have been scientifically elucidated. No studies have quantitatively analyzed changes in salivary gland function after aromatherapy in patients who have undergone RAI therapy. The aim of this study was to test the effectiveness of aromatherapy as a complementary intervention for decreasing salivary gland damage induced by RAI therapy.

## 2. Materials and Methods

### 2.1. Sample and Sampling Method

This study included 71 DTC subjects who had undergone RAI therapy at Asahikawa Medical University Hospital, Japan, between June 2014 and March 2016. The trial was a randomized clinical trial registered with the University Hospital Medical Information Network (UMIN) (ID = UMIN000013968) clinical trials registry.

All subjects had primarily undergone total thyroidectomy and had also undergone RAI therapy with a mean activity of 5.31 GBq (range, 3.70–5.55 GBq) after the withdrawal of hormone therapy for at least 2 weeks. Those who received external radiotherapy of the head and neck or additional were excluded. Subjects who had a history of salivary gland disease, who were found to have autoimmune disease, or who were taking xerostomic drugs were also excluded. Furthermore, those who could not complete the evaluations of the study protocol or who had allergies to essential oils were excluded.

The minimum sample size was calculated to be 32 subjects in each group using the formula for determining the number of samples in two independent groups and by considering a significance level of *α* = 0.05, 95% confidence level and statistical power of 80%, according to a study by Seifi et al. [18]. Considering a 20% sample loss, 40 subjects were evaluated in each group (total, 80 subjects).

### 2.2. Randomization and Allocation

Subjects (*N* = 80) were randomly assigned to one of two groups, aromatherapy (group A, *n* = 40) and control (group B, *n* = 40), by random blocking of four subjects with an allocation ratio of 1 : 1. Sequence allocation was defined using a computerized random number table. To complete the blinding of allocation, numbered bottles of the same shape and size that contained essential oil or distilled water were used. During the study, five subjects in group A were excluded because they were discharged from the hospital and due to intolerance to aroma inhalation. Four subjects in group B were excluded as they were discharged from the hospital and due to lack of cooperation. Therefore, data from 35 subjects in group A and 36 subjects in group B were analyzed.

### 2.3. Aromatherapy Intervention

Subjects in group A underwent treatment with an essential oil, which consisted of a mixture of 1.0 mL Citrus limon (primary constituents were the monoterpene hydrocarbons D-limonene and *β*-pinene; Ecology Shimanto Co., Kochi, Japan) and 0.5 mL Zingiber officinale (primary constituents were the sesquiterpene hydrocarbons zingiberene and *β*-sesquiphellandrene; Ecology Shimanto Co.). Subjects in group B inhaled distilled water as a placebo. They received an aromatic bath for 10 min before each meal for 2 weeks while they were hospitalized. A board-certified lecturer from the Japan Clinical Aromatherapy Society blended the essential oil, demonstrated the inhalation method for the subjects, and confirmed inhalation.

### 2.4. Salivary Gland Scintigraphy

For the measurement of the functional outcome of aromatherapy, subjects underwent salivary gland scintigraphy before RAI therapy and 4 days after the I-131 oral administration. Dynamic imaging of the anterior head using a dual-head gamma camera with a parallel-hole, high-energy, medium-sensitivity collimator (Millennium VG, GE Medical System, Tokyo, Japan) was performed after a bolus intravenous injection of 185 MBq (5 mCi) 99mTc-pertechnetate. Sequential dynamic images were then obtained at 1 min/frame on a 128 × 128 matrix with a zoom factor of 1.5 for 30 min. The photopeak was centered at 140 keV with a 20% window. Twenty minutes after the injection, 3 mL lemon juice (100% concentrated) was instilled into each subject's mouth through a syringe to stimulate salivary secretion.

**Figure 1:**
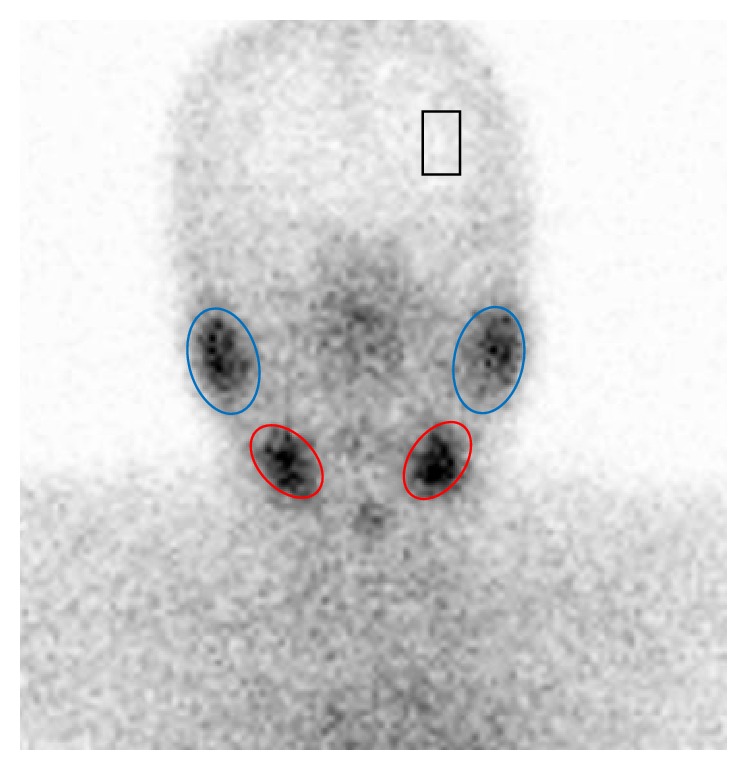
Regions of interest on the summation image as obtained by dynamic scintigraphy.

For quantitative analysis, regions of interest (ROIs) were drawn around the right and left parotid glands and right and left submandibular glands on the summation images of dynamic scintigraphy. A background ROI was placed in the temporal region (Figure 1). A time-activity curve was created for each salivary gland.

**Figure 2:**
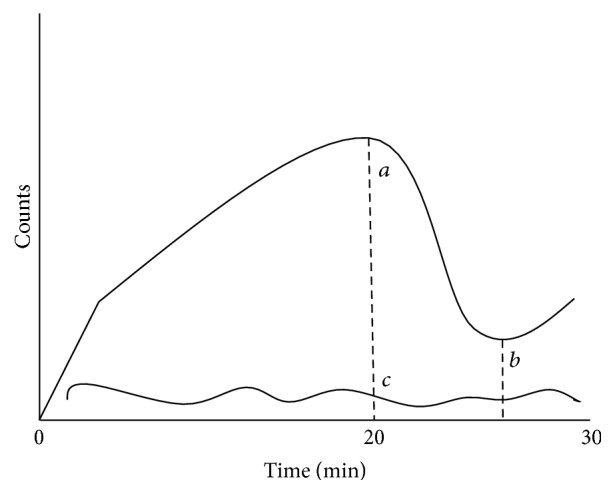
Measurement of salivary gland functions by salivary gland scintigraphy.

The following points were designated on the time-activity curve: (a) maximum count before stimulation, (b) minimum count after stimulation, (c) background count at the time of peak activity (pre- and postinjection syringes containing the 185 MBq dose were counted on the camera; counts in the postinjection syringe were corrected for decay and subtracted from counts in the preinjection syringe to determine counts injected), and (d) injected counts. The following glandular function parameters were calculated using the time-activity curves for each salivary gland: maximum accumulation ratio = (*a* − *c*)/*d* × 100 and washout ratio = [1 − (*b* − *c*)/(*a* − *c*)] × 100 (Figure 2).

ROIs for each salivary gland and background were manually defined by two senior fellows of the Japanese Society of Nuclear Medicine on the basis of a visual boundary. The maximum accumulation ratio and washout ratio were evaluated in a blinded manner and were calculated as the average of each value determined by the fellows.

### 2.5. Statistical Analysis

Data analysis was conducted using statistical software (XLSTAT2016; Addinsoft, Paris, France). All data were calculated as means ± standard errors. The normal distribution of data was analyzed with the Kolmogorov-Smirnov test. Fisher's exact test was used for categorical variables, and the Wilcoxon rank-sum test or independent samples *t*-test was used for numerical variables. A *P* value of <0.05 was considered significant.

### 2.6. Ethics

This study adhered to the Declaration of Helsinki and was approved by Asahikawa Medical University Research Ethics Committee (no. 14001). Informed consent for the secondary use of clinical information for research purposes was obtained from all study subjects.

## 3. Results

### 3.1. Homogeneity Test of Subjects

After factoring in the failure rate, data were collected from a total of 80 subjects. Nine subjects were later excluded. Thus, data from 35 subjects in group A and 36 subjects in group B were analyzed.  Table 1 shows the demographic and clinical characteristics of the 71 subjects. The pathological classification of thyroid tumors was performed according to TNM version 7 (2009) [19]. The independent samples *t*-test and Fisher's exact test showed no significant differences between study groups with respect to demographic and background variables.

### 3.2. Aromatherapy for Salivary Gland Function

**Figure 3:**
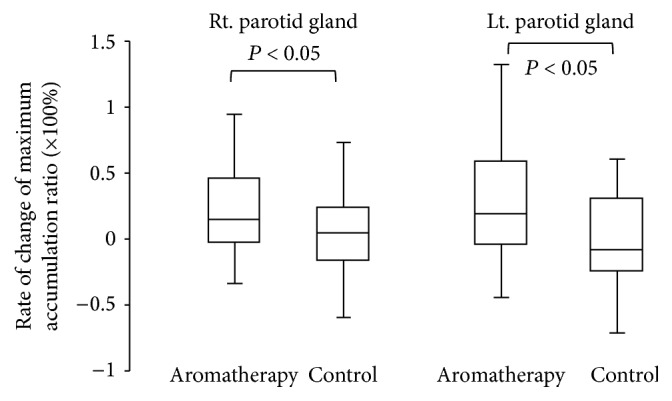
Comparison of rate of change of maximum accumulation ratio in parotid glands.

**Figure 4:**
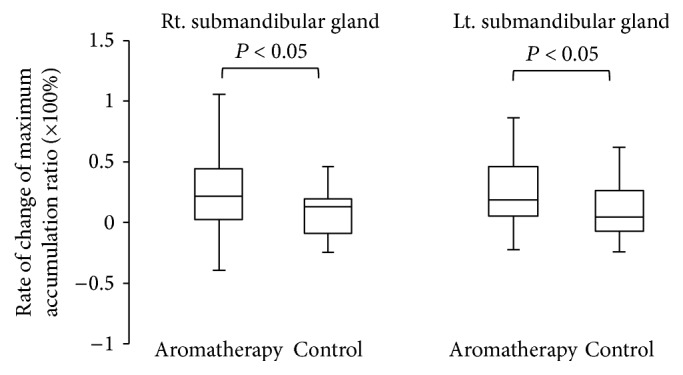
Comparison of rate of change of maximum accumulation ratio in submandibular glands.

**Figure 5:**
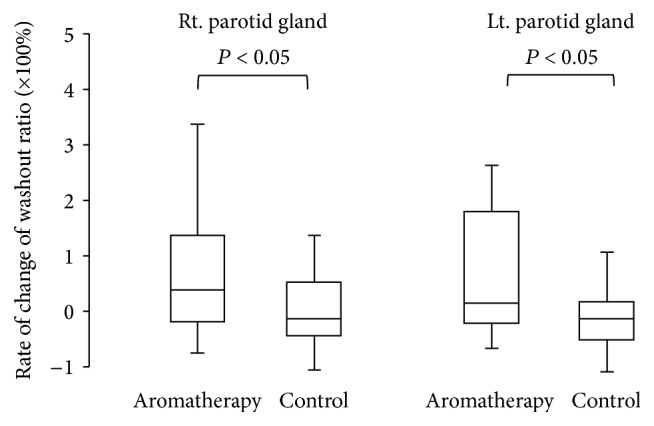
Comparison of rate of change of washout ratio in parotid glands.

**Figure 6:**
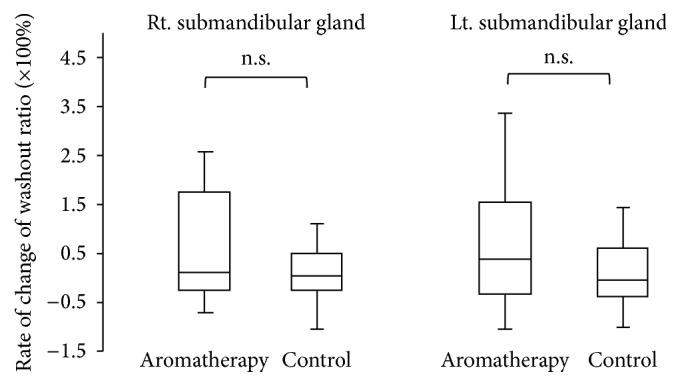
Comparison of rate of change of washout ratio in submandibular glands.

The maximum accumulation ratio as an index of salivary uptake and washout ratio as an index of salivary excretion were evaluated. The results of salivary gland scintigraphy are shown in Table 2. Compared with subjects in group B, those in group A showed a significantly higher rate of change of the maximum accumulation ratio in the parotid and submandibular glands (Figures 3 and 4, *P* < 0.05). Subjects in group A also showed a significantly increased rate of change of the washout ratio before and after the therapy in the bilateral parotid glands (Figure 5, *P* < 0.05). Although an increasing trend was observed for the submandibular glands in subjects in group A, no significant differences were noted between the groups (Figure 6).

## 4. Discussion

The presence of sodium/iodine symporters (NISs) in tissues other than the thyroid allows for the absorption of I-131 during RAI therapy. Owing to NISs, salivary glands are among the tissues with the highest absorbed levels of I-131; hence, toxic effects in salivary glands are more severe [7]. These effects are dose related, and the damage can be temporary or permanent.

In clinical practice, massage of the salivary glands and oral mucosa, exercise of the tongue, jaw, and lips, and administration of lemon candy promotes salivary gland secretion as preventive measures of disorder [20]. Aromatherapy may also be indicated for patients with decreased appetite or trismus due to functional decline associated with hormone withdrawal.

In the present study, reduced pressure steam distillation of lemon pericarp did not produce photo toxicity noted in the squeeze method. Limonene, the principal component, is an essential oil contained in large quantities in the peel of citrus fruits and is a type of monoterpene [21]. Subjects who underwent RAI therapy showed increased saliva secretion after the inhalation of an essential oil containing lemon and ginger. Lemon activates the parasympathetic and sympathetic nervous systems; therefore, this increased saliva production suggests that saliva secretion is promoted not only via the action of the autonomic nervous system but also through a conditioned reflex based on olfactory stimulation and experience.

Ginger is extracted via steam distillation of the* Z. officinale* rhizome. The principal component zingiberene is a sesquiterpene with a single ring [22] that promotes gastrointestinal mucosal protective mechanisms and salivary gland secretion. These processes regulate gastrointestinal function and have antiemetic effects [23, 24]. Zingipain is a proteolytic enzyme that assists in digestion and absorption [25], and zingerone and shogaol promote the secretion of gastric acid and regulate digestion and absorption, which initiates the activity of organs and increases appetite [24].

Furthermore, the molecules of essential oils bind with the olfactory receptors of the olfactory cilia in the nasal cavity, and sensory information is transmitted along the olfactory conduction path via the hippocampus. Simultaneously, when the memory of smelling an essential oil is stimulated, information is transmitted to the thalamus and hypothalamus, which are closely related to eating behavior. Therefore, salivary secretion may be positively influenced by aromatherapy.

Saliva is involved in taste and digestion and plays a key role in the processes of chewing and swallowing by facilitating the movement of organs in the oral cavity. It is the only digestive juice whose secretion is regulated only by the autonomic nervous system without the involvement of hormones. The results of the present study suggest that changes in salivary gland function caused by smelling the aroma of an essential oil include autonomic nervous system-mediated regulation. Saliva is secreted regardless of whether the neural activity of the sympathetic nervous system or the parasympathetic nervous system is dominant, but serous saliva is secreted in greater quantities when the activity of the parasympathetic nervous system dominates. In the present study, details such as the extent to which the autonomic nervous is involved in regulation were not elucidated. Future studies are needed to examine how autonomic nervous system activity changes owing to olfactory stimuli and verify the mechanism through which salivary secretion increases.

It has been reported that the olfactory stimulus of black pepper significantly increases serum substance P (SP) concentration and improves swallowing reflex [26]. SP exerts a variety of effects on the body but is present in the sensory branches of the vagus and glossopharyngeal nerves. It is well known that SP is retrogradely transported to the laryngopharynx and trachea and is involved in the induction of swallowing and cough reflex. Further, SP has been reported to also exert saliva-secretion-promoting effects [27–29]; therefore, considering the mechanism of saliva-secretion-promoting effects (apparently due to the olfactory stimuli of essential oil) that was revealed by the present study, further examination of the relationship with SP is needed.

The salivary glands are damaged by RAI therapy, and this damage is exacerbated as the number of therapies increases. However, because accumulated iodine is excreted after each therapy owing to saliva secretion, the degree of salivary gland impairment may be lessened. Reducing the severity of salivary gland disorders may help patients avoid therapy interruption due to adverse events. In this study, the rate of change in the washout ratio in the submandibular glands did not significantly differ between the subjects in the two groups. The parotid gland has a tendency to develop RAI sialadenitis earlier than the submandibular gland, even though NIS expression in the parotid glands does not exceed that in the submandibular gland [30]. This propensity is attributed to the higher clearance rate of salivary secretion in the submandibular glands [6, 10, 30]. We postulate that the effects of aromatherapy on submandibular gland damage are similar to those in the parotid glands; however, future studies are needed to confirm this hypothesis.

## 5. Conclusions

In the present study, we evaluated differences in salivary gland secretion function following aromatherapy treatment intervention in RAI patients. Because an amelioration of salivary gland function was observed in the present study, our results suggest the efficacy of aromatherapy in the prevention of treatment-related salivary gland disorder.

## Figures and Tables

**Table 1:** Patient characteristics.

Characteristics	Group A (*n* = 35)	Group B (*n* = 36)	*P* value
Age at diagnosis (y), mean (range)	61.6 (35–78)	59.8 (30–86)	0.61
Gender (female/male)	25/10	30/6	0.27
TNM stage			
I	2	2	0.94
II	0	0	
III	2	3	
IV A/B/C	14/0/17	12/1/18	
I-131 dose (GBq), average ± standard deviation	5.37 ± 0.20	5.24 ± 0.66	0.59

**Table 2:** Comparisons of salivary gland scintigraphic parameters by using the Wilcoxon rank-sum test.

	Group A	Group B	*P* value
Rate of change of maximum accumulation ratio [%]			
Rt. parotid gland	29.31 ± 10.65	4.66 ± 5.50	<0.05
Lt. parotid gland	47.08 ± 14.34	4.19 ± 5.98	<0.05
Rt. submandibular gland	19.56 ± 6.23	1.96 ± 3.24	<0.05
Lt. submandibular gland	13.91 ± 4.77	0.16 ± 3.86	<0.05
Rate of change of washout ratio [%]			
Rt. parotid gland	82.75 ± 38.56	18.58 ± 19.66	<0.05
Lt. parotid gland	185.73 ± 75.78	3.39 ± 11.87	<0.05
Rt. submandibular gland	60.73 ± 29.11	47.06 ± 17.96	0.45
Lt. submandibular gland	94.96 ± 33.13	18.58 ± 19.66	0.10
